# Robotic-assisted tumor enucleation versus robotic-assisted partial nephrectomy for intermediate and high complexity renal cell carcinoma: a single-institution experience

**DOI:** 10.1186/s12957-023-03060-3

**Published:** 2023-06-08

**Authors:** Kunyang Lei, Xu Wang, Zhongsheng Yang, Yuming Zhong, Yifu Liu, Ting Sun

**Affiliations:** 1 Department of Urology, the First Affiliated Hospital of Nanchang University, Nanchang, 330006 Jiangxi China; 2 Department of Pathology, the First Affiliated Hospital of Nanchang University, Nanchang, 330006 Jiangxi China; 3 Department of Urology, Ganzhou People’s Hospital, Ganzhou, 341000 Jiangxi China

**Keywords:** Complex renal cell carcinoma, RATE, RAPN, Oncological outcome

## Abstract

**Objectives:**

To compare the perioperative and oncological outcomes of robotic-assisted tumor enucleation (RATE) and robotic-assisted partial nephrectomy (RAPN) in the treatment of intermediate and high complexity renal cell carcinoma (RCC).

**Methods:**

We retrospectively collected the data of 359 patients with intermediate and high complexity RCC who underwent RATE and RAPN. The perioperative, oncological, and pathological outcomes of the two groups were compared, and univariate and multivariate analyses were used to evaluate the risk factors for warm ischemia time (WIT) > 25 min.

**Results:**

Compared with RAPN group, patients in RATE group had shorter operative time (*P* < 0.001), shorter WIT (*P* < 0.001), and less estimated blood loss (EBL) (*P* < 0.001). The decrease rate of estimated glomerular filtration rate (eGFR) in RATE group was better than that in RAPN group (*P* < 0.001). Multivariable analysis showed that RAPN and higher PADUA score were independent risk factors for *WIT* > 25 min (both *P* < 0.001). The rate of positive surgical margin was similar between the two groups, but the local recurrence rate of the RATE group was higher than that of the RAPN group (*P* = 0.027).

**Conclusions:**

RATE and RAPN have similar oncological outcomes for the treatment of intermediate and high complexity RCC. In addition, RATE was superior to RAPN in perioperative outcomes.

## Introduction

Renal cell carcinoma (RCC) is a common malignant tumor of the urinary system, and its incidence is increasing year by year [1]. With the increasing detection rate of localized RCC, nephron-sparing surgery (NSS) is becoming more and more popular. Partial nephrectomy (PN) is the removal of a renal tumor and part of the normal kidney tissue adjacent to the tumor [2]. For localized RCC, PN has similar oncological results with radical nephrectomy (RN), and it has better preserved patient renal function [3]. However, studies have shown that even if only 1 cm thickness of normal kidney tissue is removed near the tumor, renal function is still impaired. In addition, the width of resection of normal tissue adjacent to the tumor has been controversial. Studies have shown that even if the thickness of the removed normal tissue is only 1 mm, the probability of tumor recurrence does not increase [4, 5]. In addition, because more kidney tissue is preserved, the patient’s renal function is preserved to the greatest extent. Based on the above theory, some scholars have proposed tumor enucleation (TE), that is, only the tumor is removed along the capsule bluntly, without removing the macroscopic normal renal tissue, so as to maximize the preservation of renal function [6].

For the treatment of complex renal tumors, robot-assisted partial nephrectomy (RAPN) reduces the difficulty of operation and shortens the operation time and warm ischemia time (WIT), so it has higher superiority than laparoscopic partial nephrectomy (LPN). Previous studies have compared robotic-assisted tumor enucleation (RATE) with RAPN in the treatment of localized RCC [7]. However, the difference between RATE and RAPN in the treatment of intermediate and high complexity localized RCC remains unknown. Therefore, the aim of this study was to compare the perioperative and oncological outcomes of RATE and RAPN in the treatment of intermediate and high complexity RCC, thereby providing new insights into the treatment of patients with intermediate and high complexity RCC.

## Materials and methods

### Patients

We retrospectively collected the clinical data of patients with localized RCC who underwent RATE or RAPN in our hospital from June 2014 to July 2022. After excluding patients with multifocal tumors, PADUA score < 8, solitary kidney, and incomplete data, a total of 359 patients were enrolled in the study. All patients signed an informed consent form. Our study was approved by the Ethics Committee of the First Affiliated Hospital of Nanchang University.

**Fig. 1:**
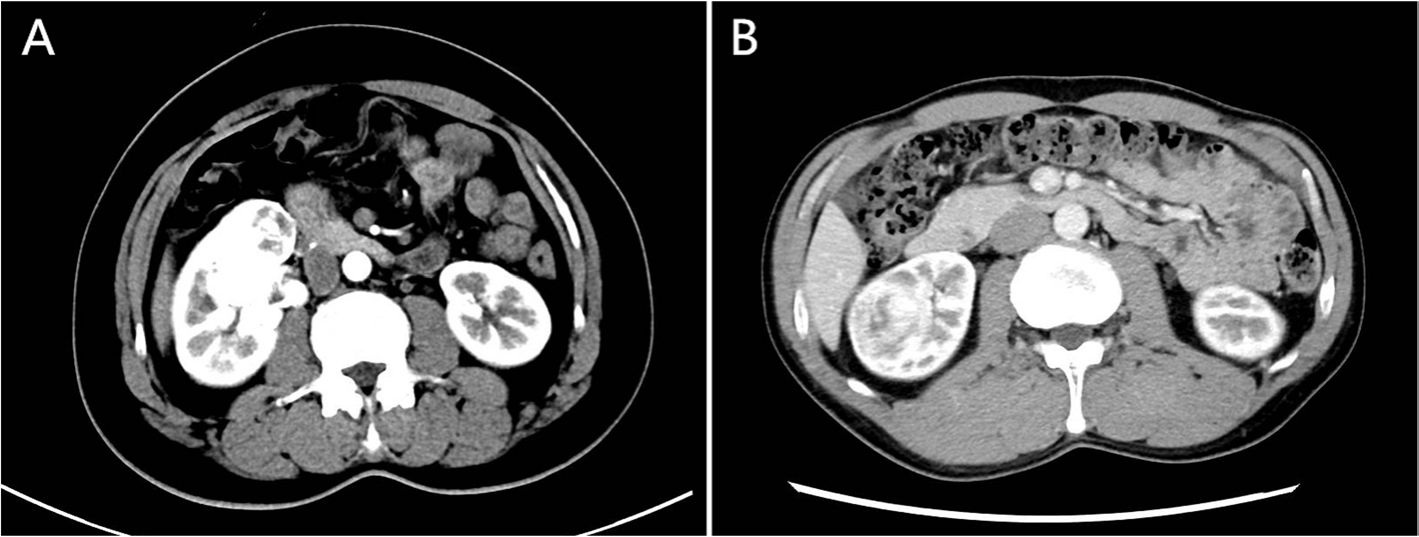
CT findings of complex renal cell carcinoma. **A** Intermediate complexity renal cell carcinoma (PADUA score: 9). **B** High complexity renal cell carcinoma (PADUA score: 11)

Data to be evaluated include patient demographics, perioperative outcomes, and oncological outcomes. The American Society of Anesthesiologists (ASA) score was used to evaluate the patient’s tolerance to anesthesia. The PADUA score was used to assess the complexity of tumors, and intermediate and high complexity tumors are defined as preoperative aspects and dimensions used for an anatomical (PADUA) scores ≥ 8 (Fig. 1) [8]. Complications were classified according to the Clavien-Dindo classification [9]. The estimated glomerular filtration rate (eGFR) was estimated by modification of diet in renal disease study group [10].

### Surgical technique

The surgical techniques of RATE and RAPN have been explained in detail in previous study [11]. In general, the RATE means that the pseudocapsule is incised at a distance of 1–2 mm from the tumor, and then the tumor is excised by blunt dissection following the natural plane between the peritumor pseudocapsule and the renal parenchyma without removing a visible rim of renal parenchyma. In contrast, RAPN included the sharp excision of the tumor and surrounding normal renal parenchyma of 0.5 to 1 cm thickness. Other than that, the two procedures are basically the same.

### Pathology

The specimens were embedded in paraffin and then sectioned and stained. Histological subtype, nuclear grade, and surgical margin of the tumor were evaluated by two pathologists. Histological subtypes were assessed according to the 2016 WHO Classification of Tumours of the Urinary System [12]. Nuclear grading was based on 2016 WHO/ISUP Grading System [13]. The criteria for a positive surgical margin is microscopically visible tumor margin with cancer cell infiltration.

### Follow-up

Follow-up was performed every 3 months during the first year after surgery and every 6 months thereafter. During the follow-up period, physical examination, hematology, renal function, and abdominal and chest imaging examinations should be evaluated. For patients who could not come to our hospital for review, the health status of the patients was assessed by telephone.

### Statistical analysis

All data in this study were statistically analyzed using IBM SPSS 26.0. Continuous variables were expressed as median and interquartile range (IQR), and comparisons between groups were performed using the Mann–Whitney *U*-test. Categorical variables were expressed as percentages, and comparisons between groups were performed using Pearson chi-square test. Univariable and multivariable logistic regression models were used to evaluate the risk factors for *WIT* > 25 min. All *P*-values were 2-sided, and a *P*-value of < 0.05 was considered statistically significant.

## Results

The preoperative characteristics of patients are summarized in Table 1. Of the 359 patients, 135 underwent RATE, and 224 underwent RAPN. Overall, there were no statistic differences between the two groups in gender, age, BMI, symptoms at diagnosis, tumor side, tumor size, ASA score ≥ 3, PADUA score, and preoperative eGFR.

**Table 1:** Preoperative characteristics of patients

Variables	RATE (*n* = 135)	RAPN (*n* = 224)	*p*-value
Age, median (IQR), year	55 (47, 63)	55 (48, 64)	0.874
Gender, *n* (%)			0.625
Male	87 (64.4)	150 (67.0)	
Female	48 (35.6)	74 (33.0)	
BMI, median (IQR), kg/m^2^	21.2 (20.1, 22.1)	21.5 (20.5, 22.0)	0.552
Symptoms at diagnosis, *n* (%)			0.793
Symptomatic	25 (18.5)	44 (19.6)	
Asymptomatic	110 (81.5)	180 (80.4)	
Tumor side, *n* (%)			0.882
Left	71 (52.6)	187 (52.1)	
Right	64 (47.4)	172 (47.9)	
Tumor size, median (IQR), cm	4.8 (4.2, 5.5)	4.5 (4.0, 5.3)	0.178
ASA score ≥ 3, *n* (%)	13 (9.6)	32 (8.9)	0.712
PADUA score, median (IQR)	8 (8.0–9.0)	8 (8.0–9.0)	0.345
Preoperative eGFR, median (IQR), mL/min/1.73 m^2^	98.0 (90.0, 108.0)	100.0 (90.5, 104.0)	0.199

*IQR*, interquartile range, *BMI*, body mass index, *ASA* American Society of Anesthesiologists, *PADUA*, preoperative aspects and dimensions used for an anatomical, *eGFR*, estimated glomerular filtration rate

In terms of perioperative outcomes, the operation time in the RATE group was shorter than that in the RAPN group (165 vs. 175 min, *P* < 0.001). WIT in RATE group was significantly shorter than that in the RAPN group (21 vs. 25 min, *P* < 0.001). In addition, estimated blood loss (EBL) was significantly reduced in the RATE group compared with the RAPN group (120 vs. 150 min, *P* < 0.001). The decrease rate of eGFR in the RATE group was lower than that in the RAPN group (8.1% vs. 13.0%, *P* < 0.001). Postoperative hospital stay and the incidence of serious complications were similar in the two groups (Table 2). To identify the risk factors leading to *WIT* > 25 min, we performed a logistic regression analysis (Table 3). Univariable analysis showed that larger tumor size, RAPN, and higher PADUA score were risk factors for WIT > 25 min (all *P* < 0.001). Multivariable analysis showed that RAPN and higher PADUA score were independent risk factors for *WIT* > 25 min (both *P* < 0.001).

**Table 2:** Perioperative outcomes

Variables	RATE (*n* = 135)	RAPN (*n* = 224)	*p*-value
Operative time, median (IQR), min	165 (150.0, 180.0)	175 (160.0, 185.0)	< 0.001
WIT, median (IQR), min	21 (18.0, 23.0)	25 (24.0, 27.0)	< 0.001
EBL, median (IQR), mL	120 (100.0, 160.0)	150 (125.0, 190.0)	< 0.001
Postoperative hospital stay, median (IQR), day	6 (6.0, 7.0)	7 (6.0, 7.0)	0.273
Clavein-Dindo grades 3–4, *n* (%)	12 (9.0)	33 (14.7)	0.116

*WIT* warm ischemia time, *EBL* estimated blood loss, *IQR* interquartile range, *RATE* robotic-assisted tumor enucleation, *RAPN* robotic-assisted partial nephrectomy

**Table 3:** Univariable and multivariable analysis for WIT > 25 min

Variables	Univariable analysis			Multivariable analysis		
	*OR*	95% *CI*	*p*-value	*OR*	95% *CI*	*p*-value
Age	0.996	0.979–1.013	0.617	1.008	0.984–1.033	0.504
Gender	0.790	0.491–1.271	0.330	0.647	0.353–1.186	0.159
BMI	1.015	0.880–1.170	0.841	1.139	0.951–1.364	0.156
Tumor side	0.995	0.683–1.551	0.982	0.857	0.499–1.535	0.642
Tumor size	2.018	1.593–2.555	< 0.001	1.391	0.903–2.144	0.135
ASA score ≥ 3	0.462	0.184–1.155	0.098	0.326	0.099–1.073	0.065
PADUA score	2.515	1.932–2.273	< 0.001	3.132	1.911–5.132	< 0.001
RATE vs. RAPN	0.127	0.068–0.239	< 0.001	23.319	9.308–58.415	< 0.001
Preoperative eGFR	0.998	0.978–1.018	0.854	0.996	0.970–1.023	0.756

*BMI* Body mass index, *ASA* American Society of Anesthesiologists, *PADUA* preoperative aspects and dimensions used for an anatomical, *eGFR* estimated glomerular filtration rate, *RATE* Robotic-assisted tumor enucleation, *RAPN* Robotic-assisted partial nephrectomy, *OR* Odds ratio, *CI* Confidence interval

Pathological results showed that there was no significant difference in tumor subtypes between the two groups (*P* = 0.413). There were 9 patients (6.7%) with Fuhrman grades 3–4 in the RATE group, which was similar to the RAPN group (6.7%, *P* = 0.991). The rate of positive surgical margin was 4.4% in RATE group and 4.9% in RAPN group, and there was no significant difference between the two groups (*P* = 0.840). The follow-up period was 45 and 46 months, respectively. During the follow-up period, 3 patients (2.2%) in the RATE group had local tumor recurrence and underwent RN after recurrence. Local recurrence occurred in 3 cases in RAPN group, and the recurrence rate was lower than that in RATE group (0.8%, *P* = 0.027). The patients also underwent RN after tumor recurrence and achieved good results (Table 4).

**Table 4:** Pathological and follow-up data

Variables	RATE (*n* = 135)	RAPN (*n* = 224)	*p*-value
Tumor subtype, *n* (%)			0.413
ccRCC	91 (67.4)	165 (73.7)	
PRCC	17 (12.6)	27 (12.1)	
ChRCC	9 (6.7)	14 (6.3)	
Other types	18 (13.3)	18 (8.0)	
Fuhrman grades 3–4, *n* (%)	9 (6.7)	15 (6.7)	0.991
Positive surgical margin, *n* (%)	6 (4.4)	11 (4.9)	0.840
Follow-up, median (IQR), month	45 (22, 63)	46 (24, 66)	0.692
Local recurrence, *n* (%)	3 (2.2)	3 (0.8)	0.027

*ccRCC*, clear cell renal cell carcinoma; *PRCC*, papillary renal cell carcinoma; *ChRCC*, chromophobe renal cell carcinoma

## Discussion

NSS, including PN, TE, and radiofrequency ablation, is considered the standard treatment for localized RCC [14]. TE is a blunt separation between the tumor and the normal kidney tissue, thereby removing the tumor intact but preserving as much of the normal kidney tissue as possible [15]. PADUA score is widely used to assess the complexity of renal tumors. Studies have shown that the higher the PADUA score, the higher the incidence of perioperative complications [16]. RATE, a combination of RANSS and TE, has been used to treat small and low complexity renal tumors. To the best of our knowledge, this study is currently the most included cases comparing RATE and RAPN in the treatment of intermediate and high complexity RCC.

Studies have shown that the operation time and WIT of RATE were shorter than those of RAPN, which was similar to the results of our findings [11]. RATE is operated in a pseudocapsule outside the tumor, thus avoiding damage to the urinary collecting system and large vessels [17]. This will undoubtedly reduce the difficulty of the operation, thus shortening the operation time. Previous studies have shown that shorter WIT and preservation of normal renal tissues as much as possible are the keys to prevent long-term renal function decline after surgery [18, 19]. Because RATE does not require additional processing of the urinary collecting system and large vessels, it shortens the WIT, which is critical for the preservation of renal function in patients. In the present study, patients in the RATE group had more renal function preserved, which was related to shorter WIT and less normal renal tissue being removed. Some studies have even reported a series of renal pedicle non-clamp or delayed clamp; however, we believe that this is not applicable to complex renal tumors [20]. Clamping the renal pedicle is necessary in the resection of intermediate or high complexity renal tumors because it can maintain a good surgical field of view, allowing the surgeon to more clearly identify the tumor boundary and complete resection of the tumor. In this study, there was less EBL in the RATE group than in the RAPN group, which is similar to the findings reported previously [21]. The presence of a pseudocapsule between the tumor and normal renal tissue, usually composed of fibrous connective tissue and inflammatory tissue, formed an avascular interface, which resulted in less EBL in the RATE group than in the RAPN group. RAPN removes part of the normal renal tissue, so patients are more likely to develop serious complications, such as urine leakage and bleeding, as has been demonstrated in previous studies [7]. This was not the case in our study, because we usually carefully suture the tumor bed during surgery, which resulted in a decrease in the incidence of urine leakage and bleeding.

Positive surgical margins are considered to be predictors of local tumor recurrence [22, 23]. In this study, the positive rate of surgical margin was similar between the RATE group and RAPN group. Some scholars believe that, compared with RAPN, the surgical margin of RATE is closer to the tumor, so its positive rate of surgical margin will be higher [24, 25]. However, other scholars have suggested that the pseudocapsule and degenerated renal parenchyma around the tumor can prevent tumor cells from invading the surrounding normal renal parenchyma, so excision along the pseudocapsule around the tumor does not increase the positive rate of surgical margins [26, 27]. In addition, studies have shown that although positive surgical margins may lead to local tumor recurrence, they do not affect the prognosis of patients [28]. In this study, although several patients had local recurrence of the tumor, there was no reduction in survival after undergoing a RN.

Our study has several limitations. First, this study is a retrospective study. Secondly, the choice of surgical methods is based on the preference of the surgeon, which may lead to selection bias. Finally, most of the cases in this study lacked long-term follow-up results.

## Conclusions

For intermediate and high complexity RCC, RATE and RAPN had similar oncological outcomes and prognosis. RATE was superior to RAPN in perioperative outcomes. In addition, RAPN and higher PADUA scores were independent risk factors for *WIT* > 25 min. Our study demonstrates the feasibility and superiority of RATE in the treatment of intermediate and high complexity RCC.

## Data Availability

The dataset used and/or analyzed during the current study are available from the corresponding author on reasonable request.
